# Variable Expression of Notch1 and Pax5 in Classical Hodgkin Lymphoma and Infection with Epstein–Barr in Pediatric Patients

**DOI:** 10.3390/microorganisms8060958

**Published:** 2020-06-26

**Authors:** Icela Palma-Lara, Ana Elena Sánchez-Aldana, Elva Jiménez-Hernández, Octavio Martínez-Villegas, Juan Carlos Núñez-Enríquez, Juan Manuel Mejía-Aranguré, Sara A. Ochoa, Juan Xicohtencatl-Cortes, Ariadnna Cruz-Córdova, Sergio Zavala-Vega, Mariana García-Jiménez, Alejandra Contreras-Ramos, José Refugio Torres-Nava, Guillermo Mora-Ramiro, José Arellano-Galindo

**Affiliations:** 1 Molecular and Cellular Morphology Laboratory, Escuela Superior de Medicina, Instituto Politécnico Nacional, Ciudad de Mexico 11340, Mexico; icelitpl@yahoo.com (I.P.-L.); anaele.san@yahoo.com.mx (A.E.S.-A.); 2 Departamento de Hematología Pediátrica, Centro Médico Nacional la Raza, Instituto Mexicano del Seguro Social, Ciudad de Mexico 02990, Mexico; elvajimenez@yahoo.com (E.J.-H.); tallo28@gmail.com (O.M.-V.); 3 Unidad de Investigación Médica en Epidemiología Clínica, Unidad Médica de Alta Especialidad Hospital de Pediatría “Dr., Silvestre Frenk Freud” Centro Médico Nacional Siglo XXI, Instituto Mexicano del Seguro Social, Ciudad de Mexico 06600, Mexico; jcarlos_nu@hotmail.com; 4 Coordinación de Investigación en Salud, Centro Médico Nacional Siglo XXI, Instituto Mexicano del Seguro Social, Ciudad de Mexico 06600, Mexico; juan.mejiaa@imss.gob.mx; 5 Laboratorio de Investigación en Bacteriología Intestinal, Hospital Infantil de Mexico Federico Gómez, Ciudad de Mexico 06720, Mexico; saraariadnah@hotmail.com (S.A.O.); juanxico@yahoo.com (J.X.-C.); ariadnnacruz@yahoo.com.mx (A.C.-C.); 6 Laboratorio de Neuropatología, Instituto Nacional de neurología Manuel Velasco Suárez, Ciudad de Mexico 14269, Mexico; sergio.zavala.vega@gmail.com; 7 Facultad de Estudios Superiores Iztacala, Universidad Nacional Autónoma de Mexico, Ciudad de Mexico 54090, Mexico; mariana._g.j@hotmail.com; 8 Biology Development Department, Hospital Infantil de Mexico Federico Gómez, Ciudad de Mexico 06720, Mexico; acora_ramos@hotmail.com; 9 Servicio de Oncohematología, Hospital Pediátrico Moctezuma, Secretaría de Salud del Gobierno de la Ciudad de Mexico 15530, Mexico; torresoncoped@live.com.mx or; 10 Laboratorio de Virología Clínica y Experimental, Unidad de Investigación en Enfermedades Infecciosas, Hospital Infantil de Mexico Federico Gómez, Ciudad de Mexico 06720, Mexico; hibridoexperimental@gmail.com

**Keywords:** Epstein–Barr virus 1, Hodgkin lymphoma, NOTCH1, PAX5

## Abstract

NOTCH1 and PAX5 participate in the proliferation and differentiation of B and T lymphocytes. Their expression can be modified by activation of NOTCH1, induced by the Epstein–Barr (EBV) viral proteins identified as LMP1 and LMP2. To identify whether PAX5, NOTCH1, and EBV latency genes participate in the oncogenic process of pediatric patients with classical Hodgkin lymphoma (cHL), the present study aimed to identify the variable expression of NOTCH1 among disease subtypes and to assess its effect on PAX5 expression. A total of 41 paraffin-embedded tissues from Mexican pediatric patients with cHL were analyzed. The expression of CD30, CD20, NOTCH1, PAX5, and LMP1 was evaluated by immunohistochemistry and immunofluorescence. EBV detection was performed by in situ hybridization. Out of all cases, 78% (32/41) of the cHL cases were EBV positive. NOTCH1 expression was detected in 78.1% (25/32) of EBV-positive cases, nodular sclerosis being the most frequent subtype (11/25, 44%). In cases where the expression of both genes was identified, double immunofluorescence assays were conducted, finding no colocalization. We found that Reed–Sternberg cells had aberrant expression compared to their cells of origin (B lymphocytes) due to the molecular mechanisms involved in the loss of expression of PAX5 and that the identification of NOTCH1 could be considered as a candidate diagnostic/prognostic marker and a therapeutic target in pediatric cHL.

## 1. Introduction

In Mexico, hematologic neoplasms represent a significant cause of death in the pediatric population [1]. Hodgkin lymphoma (HL) represents one of the most frequent types of childhood cancer. It is classified into two groups: (a) the classical Hodgkin lymphoma (cHL), which represents 95% of cases, and (b) the nodular lymphocyte-predominant Hodgkin lymphoma (NLPHL) variety, which represents around 5% of cases [2]. Additionally, the classical form can be classified into four subtypes based on the Reed–Sternberg cell (RSC) morphology and the composition of the reactive cell infiltrate observed in the lymph node biopsy specimen. These subtypes are (1) Nodular sclerosis Hodgkin lymphoma (NSHL), (2) mixed cellularity (MCHL), (3) lymphocyte-rich, and (4) lymphocyte-depleted forms (LDHL) [2]. 

It has been estimated that 30–40% of cHL cases are associated with infection of the Epstein–Barr virus (EBV). This virus has a worldwide distribution [3]; however, the frequency of cHL-EBV-positive cases varies across geographical regions and ethnic groups [4]. For instance, in some countries, the virus has been detected in 50% of Reed–Sternberg cells (RSCs), whereas in others, the frequency can reach up to 77% [5].

The inhibition of expression of specific genes participating in B cell development, such as E2A, EBF, and PAX5, has been involved in the immunophenotypic changes of Hodgkin RSCs. PAX5 is a transcription factor that participates in B lymphopoiesis; it defines the cellular fate of B cells, promoting pre-B and B cell survival signals by activating B lineage-specific genes and by repressing others from different lineages (T, myeloid). The negative regulation of PAX5 takes place during the final activated B cells’ differentiation phase. The involvement of PAX5 in other malignant B cell tumors, such as non-Hodgkin lymphomas and acute lymphoblastic leukemias, has been described as a dysregulation provoked by chromosomal translocations due to somatic hypermutation (SHM) and class-switch recombination [6,7,8]. In the T lineage, the role of PAX5 interfere on he expression of specific genes amongst the transmembrane receptor, NOTCH1 [8]. On the other hand, NOTCH1 promotes the degradation of the E47 protein, in homodimers which, together with EBF, are essentials for PAX5 transcription [9]. Evidence suggests that viral proteins such as LMP2A utilize NOTCH1 to alter the identity of B cells; furthermore, aberrant expression of NOTCH1 can interfere with the B lymphoid phenotype [10,11]. To identify whether PAX5, NOTCH1, and EBV infection participate in the oncogenic process of classical Hodgkin lymphoma (cHL), the present study aimed to identify the variable expression of NOTCH1 among disease subtypes and to assess its effect on PAX5 expression. 

## 2. Materials and Methods

### 2.1. Cases

A total of 41 paraffin-embedded tissues were obtained from patients with Hodgkin lymphoma between 2005 and 2010. They were diagnosed and treated at the Hospital Pediatrico de Moctezuma, a hospital of the Ministry of Health in Mexico City. Written informed consent for participation was signed by the parents of the children. The distribution of these subtypes was according to the WHO classification [12]. In the present study, only samples from the cHL cases (*n* = 41) were processed for further analyses. 

### 2.2. Epstein–Barr Detection

The detection of EBV was performed through the identification of the EBV-encoded small RNA (EBER) by in situ hybridization (as previously published by our group) and through the detection of LMP1 using immunohistochemistry on the RSCs [13].

### 2.3. Immunohistochemistry of Tissue Microarrays

Tissue microarrays were used to homogenize the immunohistochemistry and immunofluorescence techniques. With the help of a 5 mm buster punch, we took a sample from the region of interest from the biopsy of the lymph node, and then it was distributed in an orderly manner for the formation of a new block. Afterward, 5 µm slices were made, which were mounted on poly-l-lysine-treated slides for immunohistochemistry and immunofluorescence with their corresponding antibodies.

### 2.4. Immunostaining

The slides were labeled using monoclonal and polyclonal antibodies for NOTCH1 (Abcam Cambridge, MA, USA; catalog number: ab82573), PAX5 (Santa Cruz Biotechnology, Santa Cruz, CA, USA; catalog number SC-55515E9), and LMP-1 EVB protein (Santa Cruz, Biotechnology, Santa Cruz, CA, USA; catalog number S-71023SC clone 3H2104,a,b,c). In brief, the slides were treated with citrate buffer 1X (Antigen Retrieval Solution catalog CB910M Biocare, Pacheco, CA, USA) and the antigen recovered to 121 °C/5 min. Afterward, the endogenous peroxide was blocked with peroxide of hydrogen 3%/10 min, and the tissues were washed with PBS-Tween 20 and then incubated with a blocking reagent (Background sniper cat. BS966 Biocare medical, Pacheco, CA, USA). Primary antibodies diluted at 1:50 were added to the slides and incubated for 12 h/4 °C under a humidity chamber; then, a secondary labeled antibody was added to the first slide with streptavidin-peroxidase (Starr Trek Universal HRP Detection System, CAT STUHRP70010-KIT, Biocare, Pacheco, CA, USA). In a second slide, an anti-mouse antibody labeled with fluorescein isothiocyanate (Santa Cruz Biotechnology, Santa Cruz, CA, USA; catalog number: SC516140) was added, and slides were treated with RNAse and washed with SSC 1X buffer; then, the nucleus was stained with blue DRAQ7^TM^. A third slide was used for double fluorescence staining using anti-NOTCH and anti-PAX (1:50), the first coupled to FITC (Santa Cruz Biotechnology Company Santa Cruz, CA, USA) and the second to CY5. Each one was incubated for 1 h (the first followed by the second), treated with RNAse, and washed as described above. The fluorescence analysis was performed in a confocal Axiovert Carl Zeiss 100 M LSM 510 with a fluorescence channel of 488 and 543 nm as well as short pass filters (BP 505-530) for FITC and large pass ones (LP560) for CY5. Positive controls were used as follows: for NOTCH1, tissue from ovarian cancer was used, and for PAX5, tissue from reactive nodes was analyzed, since it is known that the expression of these genes is increased in these tissues.

### 2.5. Variable Definitions

EBV infection was considered when the viral micro-RNA EBER was detected by in situ hybridization in RSCs. Positive expression of NOTCH1 and PAX5 was considered according to the detection performed by immunofluorescence assays.

### 2.6. Statistical Analysis

Descriptive statistics were calculated to establish the frequency of distribution of NOTCH1 and PAX5 expression in EBER-positive cases and among cHL subtypes.

## 3. Results

### 3.1. EBV Detection

**Figure 1:**
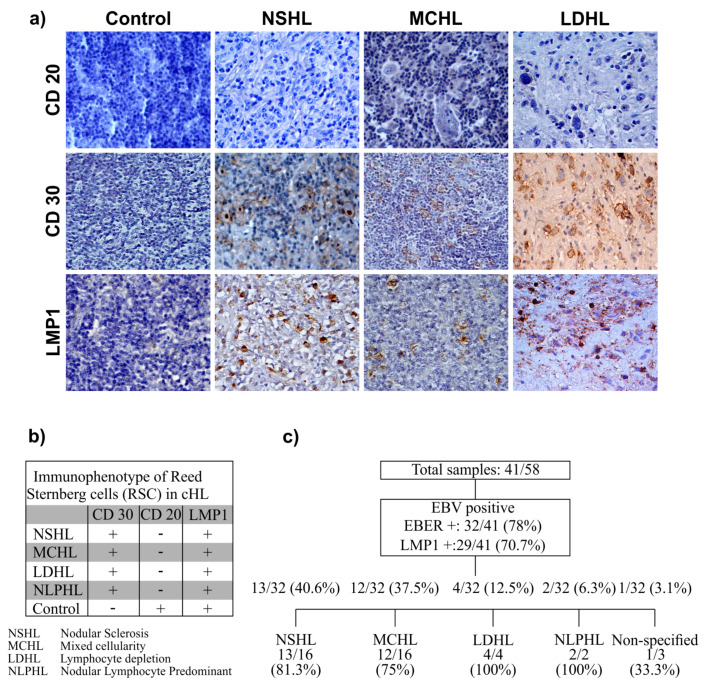
(**a**) Immunophenotype of Reed–Sternberg cells (RSC) in cHL, its different types, and positivity to LMP1 by immunohistochemistry (original magnification ×400). (**b**) The table summarizes the immunoreactivity of the immunophenotype markers (CD30 and CD20) and positivity to LMP1 in Reed–Sternberg cells in the different subtypes of cHL by immunohistochemistry in paraffin-embedded samples. (**c**) Number of cases identified with the different types of cHL positive for Epstein–Barr Virus (EBV).

A total of 32/41 (78%) tissues were EBV-EBER+ and 29/41 (70.7%) LMP-1+. All the tissues positive for LMP-1 were also positive for EBER. Nonetheless, 3/41 EBER+ were negative for LMP-1. The distribution among different cHL subtypes is displayed in Figure 1. The majority of EBV+ cases corresponded to nodular sclerosis (40.6%) and mixed cellularity (37.5%) subtypes. In three EBER+ cases, it was not possible to establish the specific subtype; however, considering all the cases analyzed, the EBV infection was detected in a range of 66–100%. The Reed–Sternberg cell count (RSCC) was high (>61 cell/microscope field) in most cases of NSHL and MCHL (the groups with more cases), with 7/13 (53.8%) for NSHL and 6/12 (50%) for MCHL; in other types, RSCC was of <20% (Table 1).

### 3.2. Age at Presentation and Immunophenotype

The age range of the study population at the time of the lymphoma diagnosis was between 0.3 and 15 years, with an arithmetic mean of 5.6 years and a standard deviation of ±4.2 years. The CD30, CD20 immunophenotype was identified (Figure 1).

### 3.3. NOTCH1 Expression

**Figure 2:**
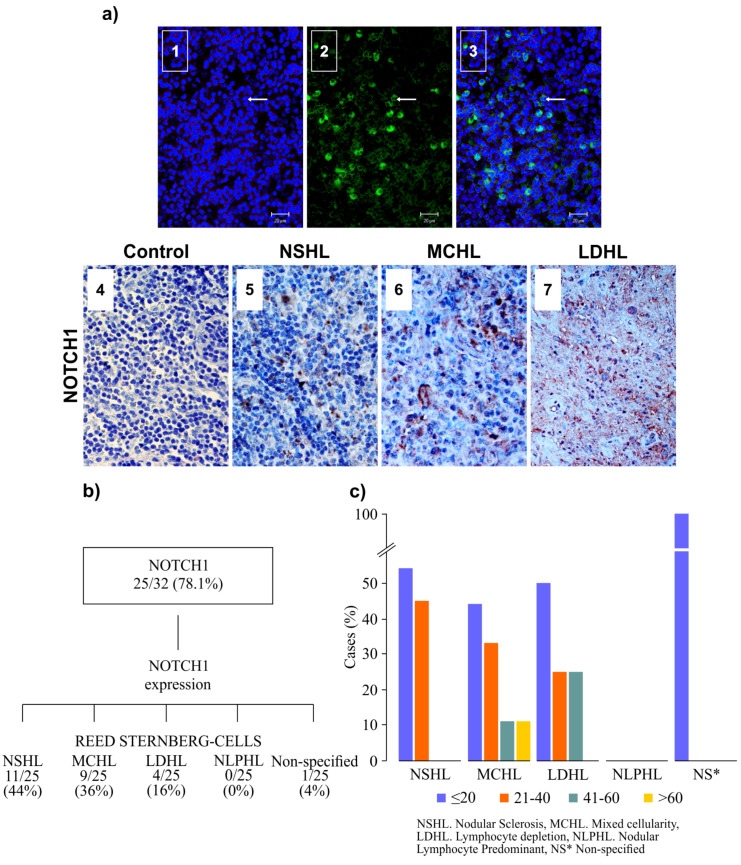
NOTCH1 expression in cHL is shown. (**a**) Immunoreactivity for NOTCH1 by multi-channel fluorescence microscopy automated image system (Applied Imaging Co.) (1–3) and by immunohistochemistry (4–7). 1. Confocal analysis labeling of cHL nuclei by DRAQ7TM (blue) in a case of NSHL; 2. NOTCH1 labeling with fluorescein, arrow (green); 3. Merge of nuclei and immunoreactivity for NOTCH1; (4–7) Immunohistochemistry for automated image system (Applied Imaging Co.): 4. Negative control for NOTCH1; 5. Immunoreactivity for NOTCH1 in NSHL; 6. Immunoreactivity for NOTCH1 in MCHL; 7. Immunoreactivity for NOTCH1 in LDHL (original magnification ×400: bars 20 µm). (**b**) Graph showing a number of cases identified with the different types of cHL positive for NOTCH1 immunoreactivity. (**c**) The graph shows the percentage of cases that expressed NOTCH1 in the different types of cHL and the range of Reed–Sternberg cells (RSCs) positive for NOTCH1 by immunohistochemistry in paraffin-embedded samples.

Of the cHL-EBV-positive patients (32/41), a total of 25/32 (78.1%) expressed NOTCH1. NSHL (*n* = 11 out of 25, 44%) and MCHL (*n* = 9 out of 25, 36%) were the main subtypes of cHL associated with NOTCH1 expression (Figure 2). When the expression of NOTCH1 was evaluated, taking into account the subtypes of cHL and the count of RSC, high expression was more frequently observed in the NSHL, MCHL, and LDHL groups with less than 40 RSCs per field (Figure 2c).

### 3.4. PAX5 Expression

**Figure 3:**
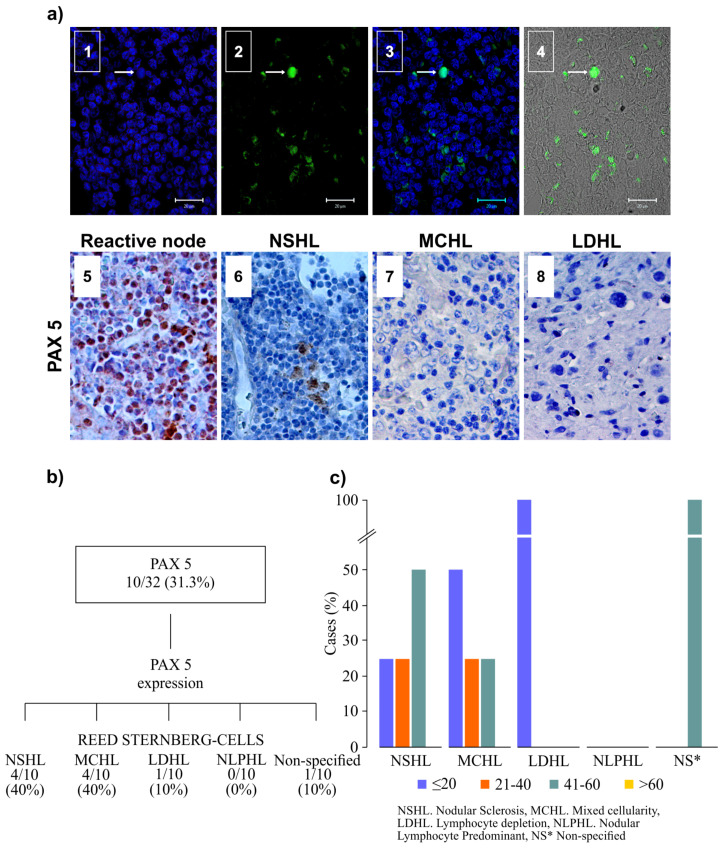
PAX5 expression in cHL. (**a**) Immunoreactivity for PAX5 by Multi-channel fluorescence microscopy automated image system (Applied Imaging Co.) (1–4) and by immunohistochemistry (5–8). 1. Confocal analysis showing the labeling of cHL nuclei by DRAQ7TM (blue) in a case of NSHL; 2. PAX5 labeling with fluorescein, arrow (green); 3. Merge of nuclei and immunoreactivity for PAX5; 4. Optical transmission showing the analyzed area of the tissue and labeling of PAX5; 5. Negative control for PAX5; 6. Immunoreactivity for PAX5 in NSHL; 7. Negative immunoreactivity for PAX5 in MCHL; 8. Negative immunoreactivity for PAX5 in LDHL (original magnification ×400: bars 20 µm). (**b**) Graph showing the number of cases identified with the different types of cHL for PAX5-positive immunoreactivity. (**c**) The graph shows the percentage of cases that expressed PAX5 in the different types of cHL and the range of Reed–Sternberg cells (RSC) positive for PAX5 by immunohistochemistry in paraffin-embedded samples.

PAX5 expression was observed in 10/32 (31.3%) of the cHL-EBV-positive patients (Figure 3a). Of these, 4/10 (40%) corresponded to the subtype NSHL, and the same percentage corresponded to the MCHL group (Figure 3b). High PAX5 expression was most frequently observed in the NSHL and MCHL groups with less than 40 RSCs per field (Figure 3c).

## 4. Discussion

PAX5 transcription factor regulates B lymphopoiesis through bimodal activity. On the one hand, it activates specific genes of B cells, and on the other hand, it inhibits those genes not associated with B lineage [14]. A NOTCH1 signaling pathway is essential for the development of the lymphoid T lineage in the thymus [15]. In the B cell lineage, the transmembrane NOTCH1 protein is involved in the maintenance of hematopoietic stem cells [16]. Usually, NOTCH1 is repressed by PAX5; however, aberrant expression of NOTCH1 interferes with the B lymphoid phenotype of neoplastic B cells in the classical Hodgkin lymphoma [11,17]. LMP2A EBV viral protein induces activation of NOTCH1, while other viral proteins such as EBNA2 interact with the RBPJK DNA binding protein responsible for mediating the intracellular binding of NOTCH1 [18]. This suggests that viral proteins can drive cellular mechanisms to the development of lymphoma. On this basis, we determined the immunoreactivity of NOTCH1 and PAX5 by immunohistochemistry, which can be used to analyze the variable expression of NOTCH1 and PAX5 in RSCs of pediatric patients diagnosed with cHL and positive for EBV infection.

NOTCH1 and PAX5 are expressed during B cell differentiation, as well as in neoplastic cells of this lineage [19,20]. Previous studies have shown aberrant expressions of NOTCH1 in lymphomas and leukemias [21]. In our study, we identified that NOTCH1 was expressed in a high proportion (78.1%) of cHL-EBV-positive patients. Despite the low number of cases, we analyzed the distribution and observed a different frequency of NOTCH1 expression among the different subtypes. Most cases were found in NSHL and MCHL subtypes, followed by LDHL and the “non-specified” group. When the expression of NOTCH1 was analyzed considering the RSC count, most of the cases had low RSC counts per field observed. These data suggest that NOTCH1 expression in cHL may have several routes by which it is increased, and that associated with the EBV infection can become more complex. Despite the need for further studies to validate these findings, our results provide evidence that NSHL and MCHL may be the subtypes most susceptible to showing aberrant expression of NOTCH1.

Regarding the frequency of PAX5 expression, it was low in the present study (31.3%) (Figure 3b). The expression of PAX5 was more frequently noted in the NSHL and LDHL groups and absent in NLPHL. Only NSHL and MCHL showed expression in three out of the four categories of RSC per field (<20, 20–40, and >60). These results suggest that, despite the low or lack of PAX5 expression in most cases, its expression is not sufficient to avoid a phenotype B in the RSCs, as has been reported [22].

It has been previously described that the loss of expression of PAX5 can convert mature B cells utilizing retro-differentiation into functional T cells [7]. It is important to emphasize that in lymphoid neoplasms, the expression of genes did not correspond to their cell lineage, and “lineage infidelity’’ makes it more difficult to diagnose or treat patients. Although PAX5 is considered a better marker than CD19 in mature lymphoid neoplasms, it should be noted that its expression is not entirely specific. The decrease in PAX5 expression could be explained by the general down-regulation of B cell-specific genes observed in Hodgkin Reed–Sternberg cells. Then, it has to be considered that PAX5 is the gene with the highest frequency of somatic mutations in patients with acute lymphoblastic leukemia, resulting in lower levels of the protein, which suggests that these molecular alterations decrease the expression of this gene in some B cell lymphomas [23]. Our results are similar to those of Adams H., et al., (2009), who performed a microarray analysis of 148 Hodgkin lymphomas and reported an immunophenotype of CD19 (3%), CD20 (30%), CD79a (11%), and PAX5 (37%) [8,23]. In addition, a previous work described that PAX5 could be a useful marker in the diagnosis of HL with atypical immunophenotypic characteristics, although in cHL, PAX5 expression may be weaker [24]. In contrast, several studies have reported that the expression of PAX5 ranges from 88–100% in patients with cHL [25,26,27,28]. However, the reason for this large range of PAX5 expression could not be clarified. It has also been stated that the specific transcriptional machinery of B cells can be altered, as well as immunoglobulin promoters, causing a decreased level or an absence of PAX5 expression. A lack of PAX5 expression in cHL is considered extremely rare [29]. On the other hand, cells can be reprogrammed by direct trans-differentiation from one lineage to another. PAX5 represses crucial receptors necessary for macrophage and T cell differentiation in which NOTCH1 is found. This explains that one of the reasons for the formation of aggressive PAX5-deficient lymphomas with a phenotype similar to B cell progenitors is probably due to a dedifferentiation process. The reason that dedifferentiated pro-B cells are susceptible to oncogenic transformation is still not well understood, although it has been hypothesized that the cause may lie in their self-renewal ability, an event similar to that of stem cells, changes in DNA methylation patterns, or chromatin remodeling [7].

Neoplastic cells of Hodgkin lymphoma called Reed–Sternberg derive from B cells of the germinal center. In recent studies, it has been demonstrated that among the pathogenic events involved in Hodgkin lymphoma are the presence of defective surface receptors, genetic silencing, expression of tumor necrosis receptor-associated factors, nuclear factor-kB, signal transducer, activation of transcription and cytokine pathways, and expression of cellular FLICE inhibitory protein with inhibition of caspase activities, which cause diverse apoptotic and proliferative effects. [14]. The activation of NOTCH1 in RSCs may be favored by changes in the expression of transcription factors specific to B cells, and therefore, it has been proposed to decrease its regulation [30].

In some cases, utilizing immunofluorescence, we observed that the expression of PAX5 and NOTCH1 occurred simultaneously, which confused the regulation that PAX5 has on NOTCH1. If PAX5 is expressed, it is assumed that the expression of NOTCH1 is inhibited; then, it was necessary to identify if both proteins were expressed in the same RS cell. For this purpose, double immunodetection was performed, finding that there was no nuclear colocalization of the proteins studied. With this, we demonstrated that although PAX5 expression occurred, it was not sufficient to inhibit NOTCH1 expression and that the translation of the protein is inhibited by different mechanisms, such as aberrant activation of the NOTCH pathway, which is a regulator of the activation of the NF-kB signaling pathway and has anti-apoptotic activity in RS cells [28]. Additionally, at the same time, it activates the NOTCH1 receptor, which inhibits the expression of B genes.

The subtype with the highest positivity to the Epstein–Barr virus was nodular sclerosis, a finding that differs from other reports in the literature [30]. Studies on the incidence of the virus in cHL have been carried out in populations from developed countries, which allow us to assume that the infectivity behavior is different between developed and developing countries [31,32]. The study of the association between the genes expressed by EBV and PAX5 and NOTCH1 genes is vital due to their association with the maintenance, transformation, and proliferation of RS cells.

The identification of EBERs in cHL was noted in 78% of the cases (30/41), which is higher than that reported by other authors [33]. The subtype with lymphocytic predominance was the one that presented greater positivity, which may be related to the amount of cell infiltrate, a microenvironment favorable for the development and maintenance of an aberrant immunophenotype in the RSC [34]. Studies carried out in different countries indicate that in 30–40% of cases, cHL has been associated with the Epstein–Barr virus (EBV) infection. The virus can remain in the latency, quiescent, or latency 0 phase in lymphocytes after a primary infection and depends on the balance of the immune response that can go into latency phase I, II, or III and expresses genes involved in cell transformation, proliferation, and immortalization; therefore, taking into account the socioeconomic factor in patients with cHL is very important. Although it was not the objective of this study, it is important to identify the participation of genes expressed by the virus, such as latent membrane protein 1 (LMP1), where certain variations have been associated with a poor prognosis [31,35].

Few studies carried out on samples of pediatric patients diagnosed with cHL have identified PAX5 expression and EBV co-infection. To our knowledge, there are no reports on the expression of NOTCH1 in patients diagnosed with lymphoma or associated with EBV in the Mexican pediatric population. It is important to highlight that previous studies regarding the participation of NOTCH1 in tumor development of cHL have been carried out in animal models and/or cancer cell lines.

These results are important since cHL and Epstein–Barr virus infection present different characteristics in different regions of the world, and we showed an analysis of these characteristics in the Mexican population. We concluded that the immunological status, as well as the identification of the Epstein–Barr virus, is a risk factor to take into account in the prognosis and treatment of these patients. We propose NOTCH1 as a potential diagnostic and prognostic marker and a therapeutic target to consider in pediatric cHL.

## Figures and Tables

**Table 1:** Distribution of Epstein–Barr virus infection and Reed–Sternberg cell counts across cHL subtypes.

Subtype	Total cHL Cases	EBV Status		Reed–Sternberg Cell Counts			
		Negative	Positive				
		*n* = 9	*n* = 32	<20	21–40	41–60	>61
**NSHL**	16	3	13	7	0	0	6
**MCHL**	16	4	12	6	2	0	4
**LDHL**	4	0	4	0	0	1	3
**NLPHL**	2	0	2	0	0	0	2
**Not Specified**	3	2	1	1	0	0	0

NSHL, nodular sclerosis; MCHL, mixed cellularity; LDHL, lymphocyte depletion; NLPHL, nodular lymphocyte predominant.
